# A Critical Appraisal of the Quality of Glioma Imaging Guidelines Using the AGREE II Tool: A EuroAIM Initiative

**DOI:** 10.3389/fonc.2019.00472

**Published:** 2019-06-07

**Authors:** Valeria Romeo, Arnaldo Stanzione, Lorenzo Ugga, Renato Cuocolo, Sirio Cocozza, Evangelia Ioannidou, Arturo Brunetti, Sotirios Bisdas

**Affiliations:** ^1^Department of Advanced Biomedical Sciences, University of Naples “Federico II”, Naples, Italy; ^2^Medical School, University of Ioannina, Ioannina, Greece; ^3^Department of Neuroradiology, The National Hospital for Neurology and Neurosurgery, University College London NHS Foundation Trust, London, United Kingdom; ^4^Department of Brain Repair and Rehabilitation, Institute of Neurology, University College London, London, United Kingdom

**Keywords:** AGREE II, glioma, imaging, guidelines, evidence-based medicine

## Abstract

**Background:** Following the EuroAIM initiative to assess the quality of medical imaging guidelines by using the Appraisal of Guidelines for Research and Evaluation (AGREE) II instrument, we aimed to evaluate the quality of the current imaging guidelines in patients with gliomas.

**Methods:** A literature search was conducted to identify eligible imaging guidelines considered in the management of adult patients with gliomas. The selected guidelines were evaluated using the AGREE II instrument by four independent appraisers. The agreement among the four appraisers was estimated using the intraclass correlation coefficient (ICC) analysis.

**Results:** Seven guidelines were selected for the appraisal. Six out of the seven guidelines showed an average level of quality with only one showing a low quality. The highest scores were found in Domain 1 “Scope and purpose” (mean score = 81.2%) and Domain 4 “Clarity of presentation” (mean score = 77.6%). The remaining domains showed a low level of quality and, in particular, Domain 5 “Applicability” was the most critical with a mean score of 41.7%, mainly related to a minor attention to barriers and facilitators as well as costs and resources implications of applying the guidelines. The ICC analysis showed a very good agreement among the four appraisers with ICC values ranging from 0.907 to 0.993.

**Conclusions:** The available guidelines on glioma imaging emerged as of average quality according to the AGREE II tool analysis. Based on these results, further efforts should be made in order to involve different professional bodies and stakeholders and increase patient and public involvement in any future guideline drafting as well as to improve the applicability of these guidelines into the clinical practice.

## Introduction

Malignant primary brain tumors still represent one of the most difficult cancers to treat with a rather low 5 year overall survival (1). Among these, glioma constitutes the largest subgroup with high grade-glioma, specifically glioblastoma, accounting for almost 50% of cases (2). Diagnostic imaging, particularly magnetic resonance imaging (MRI), plays a fundamental role in diagnosis, staging and follow-up of glioma patients (3, 4). Considering the very poor prognosis of such patients and the lack of an effective treatment, especially for recurrent disease, the patient management is very demanding whereas major endeavors are constantly made to develop more effective drug treatments (5, 6) and sensitive methods for early tumor detection, in particular recurrent disease as it appears crucial for prolonging survival. In this perspective, imaging and especially MRI make a substantial contribution to the assessment of response to treatment using conventional and advanced techniques that probe the tumor biology (7). The possibility to leverage the efforts by conducting multicenter studies in different research and clinical domains (e.g., treatment trials, identification of diagnostic, and prognostic imaging biomarkers) necessitates a standardization of the imaging protocols, especially in terms of clinical indications and acquisition techniques. To achieve a reasonable level of standardization, diagnostic imaging guidelines covering clinical indications, acquisition protocols, and technical details have been previously realized. However, the reliability of clinical practice guidelines has been questioned and the proposed recommendation statements should be rather judged based on the methodological rigor followed in their drafting process. In order to assess the quality of guidelines, several useful tools have been proposed (8). In particular, the updated Appraisal of Guidelines for Research & Evaluation version 2.0 (AGREE II) (9, 10), first established in 1998, is the most comprehensively validated and has been widely adopted for the quality assessment of clinical practice guidelines (11). A recent initiative to assess meticulously the quality of the current imaging guidelines has been promoted by the European Network for the Assessment of Imaging in Medicine (EuroAIM), founded by the European Institute for Biomedical Imaging Research (EIBIR) (12). First evaluations conducted in this matter revealed that the quality of imaging guideline is heterogeneous, ranging from low to high levels (13–16). In the context of the EuroAIM initiative, we aimed to evaluate the quality of the existing guidelines on the role of imaging in glioma patients.

## Materials and Methods

### Literature Search

Between October and November 2018, an exhaustive literature search was conducted on PubMed using MeSH and non-MeSH terms with and without customizing the search for “Consensus Development Conference,” “Guideline,” “Clinical Practice Guideline,” and “Government Document.” The following terms and their expansions were entered: “glioma,” “neoplasms,” “brain tumors,” “guideline,” “practice guideline,” “recommendations, health planning,” “official positions,” “diagnostic imaging,” “imaging.” Similarly, EMBASE, Scopus, Wiley Online Library and Google, including gray literature sources, were also searched. The search was focused on the most up-to-date version of the identified guidelines. Inclusion criteria were: (1) guidelines focused on the role of imaging in the management of primary brain tumors and specifically gliomas; (2) guidelines dealing with the adult population; and (3) papers with available English full text. Exclusion criteria were the following: (1) guidelines not developed under the auspices of recognized professional institutions, associations, and/or working groups; (2) clinical practice guidelines in which imaging was included in a wider, rather abstract context (e.g., guidelines dealing with cancer clinical management and treatment); (3) guidelines not dealing with the major imaging techniques employed for the assessment of gliomas, particularly MRI.

### Guideline Evaluation

Selected papers were evaluated by four independent radiologists (VR, AS, LU, RC) with 6 to 9 years of clinical expertise and research in a university hospital setting. The appraisers used the AGREE II instrument (http://www.agreetrust.org/), made of six quality domains, each “…capturing a unique dimension of guideline quality,” and including a total of 23 key items (9). Specifically: domain 1 “Scope and Purpose” includes items from 1 to 3; domain 2 “Stakeholder Involvement” comprises items from 4 to 6; domain 3 “Rigor of Development” provides items from 7 to 14; domain 4 “Clarity of presentation” contains items from 15 to 17; domain 5 “Applicability” covers items from 18 to 21; and domain 6 “Editorial Independence” includes items from 21 to 22. Domains and items are summarized in Table 1. Each item is rated on a 7-point scale, ranging from “strongly disagree” (score = 1), to “strongly agree” (score = 7). Finally, an Overall Assessment section is provided to summarize in a comprehensive way the quality of the guideline. Each appraiser was asked to assign a score to each item and to the Overall Assessment section as well as to indicate whether he/she would recommend the use of the guideline in clinical practice. Whereas they had previous exposure to the AGREE II tool (15), the appraisers also carried out the freely available online training tool consisting of an overview tutorial and a practice exercise (17).

**Table 1:** AGREE II domains and items (9).

**DOMAIN 1. SCOPE AND PURPOSE**
**Item 1:** the overall objective(s) of the guideline is (are) specifically described
**Item 2:** the health question(s) covered by the guideline is (are) specifically described
**Item 3:** the population (patients, public, etc.) to whom the guideline is meant to apply is specifically described
**DOMAIN 2. STAKEHOLDER INVOLVEMENT**
**Item 4:** the guideline development group includes individuals from all the relevant professional groups
**Item 5:** the views and preferences of the target population (patients, public, etc.) have been sought
**Item 6:** the target users of the guideline are clearly defined
**DOMAIN 3. RIGOR OF DEVELOPMENT**
**Item 7:** systematic methods were used to search for evidence
**Item 8:** the criteria for selecting the evidence are clearly described
**Item 9:** the strengths and limitations of the body of evidence are clearly described
**Item 10:** the methods for formulating the recommendations are clearly described
**Item 11:** the health benefits, side effects, and risks have been considered in formulating the recommendations
**Item 12:** there is an explicit link between the recommendations and the supporting evidence
**Item 13:** the guideline has been externally reviewed by experts prior to its publication
**Item 14:** a procedure for updating the guideline is provided
**DOMAIN 4. CLARITY OF PRESENTATION**
**Item 15:** the recommendations are specific and unambiguous
**Item 16:** the different options for management of the condition or health issue are clearly presented
**Item 17:** key recommendations are easily identifiable
**DOMAIN 5. APPLICABILITY**
**Item 18:** the guideline describes facilitators and barriers to its application
**Item 19:** the guideline provides advice and/or tools on how the recommendations can be put into practice
**Item 20:** the potential resource implications of applying the recommendations have been considered
**Item 21:** the guideline presents monitoring and/or auditing criteria
**DOMAIN 6. EDITORIAL INDEPENDENCE**
**Item 22:** the views of the funding body have not influenced the content of the guideline
**Item 23:** competing interests of guideline development group members have been recorded and addressed

### Quality Assessment

Following the AGREE II manual instructions, domain scores were “…calculated by summing up all the scores of the individual items in a domain and by scaling the total as a percentage of the maximum possible score for that domain” (17). Guideline overall quality was considered “high” when 5 or more domains scored more than 60%, “average” when 3 or 4 domains scored more than 60%, and “low” when no more than two domains scored more than 60%, as previously performed (13–16). Mean scores ± standard deviations of each guideline were then calculated. Domain overall quality was assessed by calculating the mean scores of each domain being considered as good (≥80%), acceptable (60–79.9%), low (40–59.9%), or very low (<40%).

### Statistical Analysis

The level of agreement among the four appraisers was assessed using the intraclass correlation coefficient (ICC) analysis and rated as: poor (ICC ≤ 0.20); fair (ICC from 0.21 to 0.40); moderate (ICC from 0.41 to 0.60); good (ICC from 0.61 to 0.80); and very good (ICC ≥ 0.81) (13–16). Scores collection and calculation as well as the statistical analysis were performed by an independent reviewer (SC) with 9 years of experience in scientific research and biostatistics.

## Results

### Literature Search and Guidelines Selection

**Figure 1:**
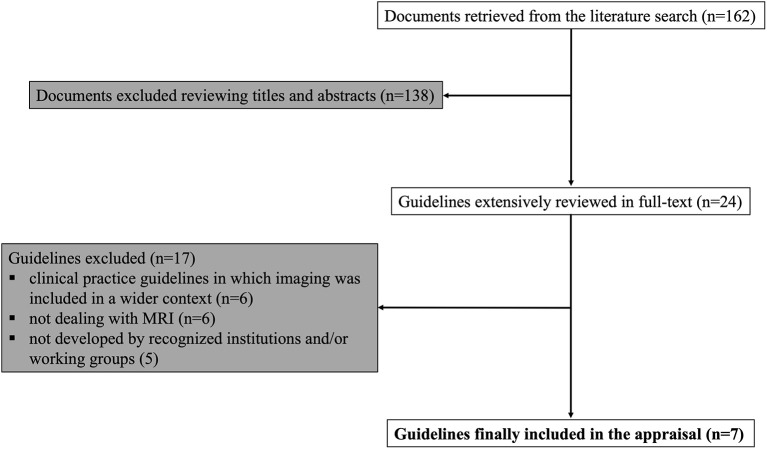
Guideline selection process.

The literature search returned 162 records. The majority of the retrieved papers was excluded after the evaluation of title and abstract, with 29 remaining articles extensively reviewed in full-text and 7 guidelines finally eligible for the appraisal process (18–24). A flow-chart of the guideline selection process is illustrated in Figure 1. Details of the selected recommendation papers are reported in Table 2.

**Table 2:** Details of the selected guidelines.

**Title**	**Year**	**Author/Organization**	**Source**	**Topic**
Recommendations for cross-sectional imaging in cancer management, Second edition-Tumors of the brain (18)	2014	Byrne et al. Royal College of Radiologists	www.rcr.ac.uk	Imaging recommendations for the management of brain tumors
Consensus recommendations for a standardized Brain Tumor Imaging Protocol in clinical trials (19)	2015	Ellingson et al. US Food and Drug Administration, National Cancer Institute	Neuro-Oncology	Recommendations for a standardized MRI protocol for the assessment of glioblastoma
MR Imaging of Neoplastic Central Nervous System Lesions: Review and Recommendations for Current Practice (20)	2012	Essig et al. Meeting expert at the “Improving Patient Management by optimizing MR Imaging of Neoplastic CNS Lesions” meeting, Zurich, 2010	American Journal of Neuroradiology	Imaging recommendations for the assessment of CNS lesions
The role of imaging in the management of adults with diffuse low-grade glioma—A systematic review and evidence-based clinical practice guideline (21)	2015	Fouke et al. Supported by the AANS/CNS Joint Guidelines Committee	Journal of Neuro-Oncology	Imaging recommendations for the assessment of diffuse low-grade glioma
Neuroradiological assessment of newly diagnosed glioblastoma (22)	2008	Mukundan et al. Supported by the AANS/CNS Joint Guidelines Committee	Journal of Neuro-Oncology	Imaging recommendations for the assessment of glioblastoma
The role of imaging in the management of progressive glioblastoma- A systematic review and evidence-based clinical practice guideline (23)	2014	Ryken et al. Supported by the AANS/CNS Joint Guidelines Committee	Journal of Neuro-Oncology	Imaging recommendations for the assessment of progressive glioblastoma
Glioma imaging in Europe: A survey of 220 centres and recommendations for best clinical practice (24)	2018	Thust et al. Endorsed by the European Society of Neuroradiology and the European Organization for Research and Treatment of Cancer	European Radiology	Clinical practice recommendations for glioma conventional and advanced MRI protocol

### Statistical Analysis

The ICC analysis showed a very good agreement among the four appraisers with values ranging from 0.907 to 0.993; the ICC scores with their 95% confidence intervals are reported in Table 3.

**Table 3:** Results of the Intraclass correlation coefficient analysis.

**References**	**ICC**	**95% CI**
Byrne et al. (18)	0.907	0.663–0.986
Ellingson et al. (19)	0.977	0.920–0.986
Essig et al. (20)	0.982	0.937–0.997
Fouke et al. (21)	0.992	0.970–0.999
Mukundan et al. (22)	0.992	0.972–0.999
Ryken et al. (23)	0.991	0.969–0.999
Thust et al. (24)	0.993	0.973–0.999

*ICC, intraclass correlation coefficient; CI, confidence interval*.

### Guideline Scores

According to the AGREE II tool, six out of seven guidelines showed an “average” quality with one guideline demonstrating “low” quality. The highest domain scores were found in Domain 1 “Scope and purpose” (mean score = 81.2%) indicating good quality, followed by Domain 4 “Clarity of presentation” (mean score = 77.6%) suggesting an acceptable quality. The remaining domains showed a low level of quality and in particular Domain 5 “Applicability” was the most critical with a mean score of 41.7%. Similarly, Domain 2 “Stakeholder involvement,” Domain 3 “Rigor of development” and Domain 6 “Editorial independence” were considered of low quality achieving mean scores of 52, 55.1, and 58.9%, respectively.

**Figure 2:**
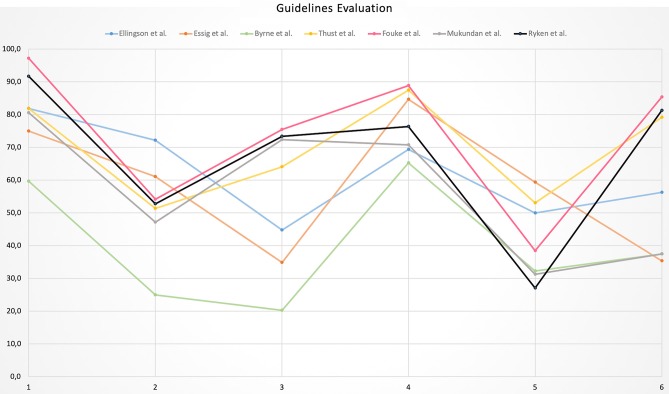
Line chart illustrating the overall domain scores of each guideline.

The highest variability in domain scores was observed in Domain 3 “Rigor of development” and Domain 6 “Editorial independence” with a SD of 21.8 and 22.7%, respectively, while the lowest variability was found in Domain 4 “Clarity of presentation” with SD of 9.5%. In the remaining domains, the variability ranged from 12 to 14.4%. All domains and guidelines scores are shown in Table 4 and Figure 2. Detailed scores of each guideline are reported in the Supplementary Materials.

**Table 4:** Results of the AGREE II-based guidelines evaluation.

**Guideline**	**Domain 1**	**Domain 2**	**Domain 3**	**Domain 4**	**Domain 5**	**Domain 6**	**Mean score**	**Guideline Overall Quality**
Byrne et al.	59.7	25.0	20.3	65.3^*^	32.3	37.5	40.0	Low
Ellingson et al.	81.9^*^	72.2^*^	44.8	69.4^*^	50.0	56.3	62.4	Average
Essig et al.	75.0^*^	61.1^*^	34.9	84.7^*^	59.4	35.4	58.4	Average
Fouke et al.	97.2^*^	54.2	75.5^*^	88.9^*^	38.5	85.4^*^	73.3	Average
Mukundan et al.	80.6^*^	47.2	72.4^*^	70.8^*^	31.3	37.5	56.6	Average
Ryken et al.	91.7^*^	52.8	73.4^*^	76.4^*^	27.1	81.3^*^	67.1	Average
Thust et al.	81.9^*^	51.4	64.1^*^	87.5^*^	53.1	79.2^*^	69.5	Average
Mean Score	81.2	52.0	55.1	77.6	41.7	58.9		
SD	12.0	14.4	21.8	9.5	12.5	22.7		
Domain Overall Quality	Good	Low	Low	Acceptable	Low	Low		

*Scores are expressed as percentages; SD, standard deviation*;

* *Domain scoring >60%. Guideline Overall quality was defined “high” when 5 or more domains scored >60%, “average” when 3 or 4 domains scored >60%, “low” when ≤2 domains scored >60%. Domain Overall quality was defined good when ≥80%; acceptable when = 60–79%; low when = 40–59%; very low when <40%*.

## Discussion

Overall, the current imaging guidelines for the management of glioma patients showed an intermediate level of quality according to the AGREE II analytical approach. In detail, six out of the seven guidelines showed an average level of quality with only one revealing low quality.

### Domain Scores

Domain 1 “Scope and purpose” and Domain 4 “Clarity of presentation” presented with the highest scores as they are primarily taken into account by guideline developers when defining the objectives and convey the recommendations. Domain 4 was the only one performing higher than 60% in all investigated guidelines. The remaining domains were judged with lower mean scores, ranging from 41.7% (Domain 5) to 58.9% (Domain 6). In particular, Domain 2 “Stakeholder involvement” performed poorly (mean score = 52%) as not all relevant professional groups (e.g., medical and/or radiation oncologist) were involved in the guideline drafting. In almost all cases, authors consisted of radiologists along with neuro-surgeons. Moreover, the views and preferences of the target patient group were not considered e.g., in terms of experiences and expectations. Although this issue may appear unwonted and not customary for medical/radiological guidelines, the AGREE II tool provides suggestions about how to facilitate patient and public involvement (e.g., by prior conferring with patients to understand main issues, using interviews or literature review on their preferences or by stakeholder's external review on the draft). Of note, target users have been scarcely specified; this is an issue that could be easily addressed by clearly indicating which professionals are meant to use the guideline.

Domain 3 “Rigor of development” assesses the methodology by which the guideline is elaborated and unfortunately obtained a low mean score (55.1%), ranging from 20.3 to 73.4% with a SD of 21.8%. The high variability discerned is due to the opacity in the methodology employed for evidence search and evaluation, enabled usually through the performance of systematic literature reviews. Only Thust et al. specifically discussed the possible methodological limitations (24). Furthermore, methods for formulating the recommendations were not always clearly named and structured techniques (e.g., the Delphi method) to reach a final consensus were not used. The most critical results were presented in Domain 5 “Applicability,” in which none of the guidelines achieved a score higher than 60%. It should be noted that issues addressed in this Domain are conventionally difficult to be considered given that resources and costs are heterogeneous among different countries and national healthcare systems. This domain also contains very specific criteria, such as the inclusion of dedicated sections to provide solutions to barrier analysis, tools to capitalize on guideline facilitators or methods by which the cost information was sought. Finally, Domain 6 “Editorial independence,” even if showing a low-quality mean score (58.9%), did not emerge as critical as occurred in previous AGREE-II evaluations (13, 14). In almost all papers, conflict of interests and funding disclosures have been stated, as the majority of guidelines is published on peer-reviewed journals, which oblige for such statements. However, in none guideline the declaration “the views of the funding body have not influenced the content of the guideline” was included.

### Considerations

General remarks can be made in light of the present appraisal, especially regarding the overall average scores of the evaluated guidelines, with none of them fulfilling high quality. While in certain guidelines the recommendations were well-underpinned by a rigorous systematic review of literature, the recommendations were disadvantaged by lacking contributions from international expert panels, other disciplines-of-interest and considerations for the routine application of the recommended procedures. On the other hand, European guidelines did not immerse in detail for the evidence search and synthesis methodology. It is worth mentioning that 4 out of 7 guidelines though close to reach a final “high” score, having each 4 domains scoring higher than 60%, they overall scored moderately as Domain 2 “Stakeholder involvement” and Domain 5 “Applicability” performed poorly. Thus, emphasizing to these domains will improve dramatically the future guidelines quality. The results of the current guidelines appraisal are better than those of a previous AGREE II evaluation of clinical practice and management guidelines in glioma patients (25). In the previous evaluation, tangible concerns were voiced for Domain 2 “Stakeholder involvement,” Domain 3 “Rigor of development,” Domain 5 “Applicability,” and Domain 6 “Editorial independence.” A further AGREE II evaluation of the clinical practice guidelines for rehabilitation on brain tumor patients also showed moderate quality results (26). Furthermore, an improvement in terms of guidelines quality over time seems to emerge from our analysis. Based on the aforementioned results, the quality of future imaging guidelines in gliomas could be further improved by summarizing the key evidence elements derived from literature review and expert consultation and to report them close to the final recommendations. Data related to any guideline external review and update process should also be provided.

### Limitations

The heterogeneity of the selected guidelines, dealing either with the definition of a standardized MRI protocol or with clinical indications of other than MRI techniques, pose an inherent limitation in our evaluation. We attempted to mitigate this risk by a universal and robust appraising tool as the AGREE II domains; we acknowledge, however, that the AGREE II instrument does not directly assess the quality of the guideline content (8). Furthermore, it is sensible that guidelines aiming to assess the role of imaging in the management of glioma patients might differ in terms of tumor sub-types (i.e., low- vs. high-grade], overall setting or even be published as appendices in wider clinical guidelines. This makes difficult a broad-based acceptance, an obvious finding in the paper of Thust et al. (24), who probed the adherence of European centers to the “mainstay” glioma MRI protocol proposed by Ellingson et al. (19). A further limitation in our study might be the exclusion of guidelines in non-English language. Finally, while initial evidence suggests to weight the domain scores for the overall quality assessment (27), we decided not to embrace this approach. Nevertheless, the possibility to prioritize one domain over the other could be considered in future AGREE II appraisals.

## Conclusions

The existing guidelines on the role of imaging in glioma patients showed an overall intermediate level of quality according to the AGREE II tool evaluation. The fairly high number of available guidelines highlights the profound interest of the oncological and radiological communities to significantly improve the management in terms of clinical indications, protocol appropriateness, and acquisition techniques. In this perspective, issues and suggestions transpired from this appraisal could be taken into account to improve the quality of imaging guidelines in neuro-oncology.

## Contribution to the Field Statement

The quality of imaging guidelines in terms of methodological rigor has been recently questioned and found to be heterogeneous, thus potentially affecting the reliability of guidelines themselves. The use of imaging guidelines is crucial for the assessment of glioma patients, especially in the context of multicenter studies and clinical trials for new drugs development and the assessment of response to treatment. We therefore joined a recent initiative of the European Network for the Assessment of Imaging in Medicine (EuroAIM) and assessed the quality of imaging guidelines focused on glioma using the Appraisal of Guidelines for Research & Evaluation version 2.0 (AGREE II) tool. According to our results, existing guidelines on glioma imaging emerged as of average quality. We also provided suggestions to further increase the quality of future guidelines on glioma imaging on the basis of the raised criticisms.

## Author Contributions

VR, SC, and EI performed the literature search. VR, AS, LU, and RC evaluated the guidelines. SC provided the statistical analysis. The manuscript was drafted by VR, AS, LU, RC, SC, and AB. Data curation was carried out by VR and SC. Critical revision was made by AB and SB. SB was responsible for project administration, study conception and design. All authors revised and approved the manuscript.

### Conflict of Interest Statement

The authors declare that the research was conducted in the absence of any commercial or financial relationships that could be construed as a potential conflict of interest.

## References

[1] Lamborn KR. Prognostic factors for survival of patients with glioblastoma: recursive partitioning analysis. Neuro Oncol. (2004) 6:227–35. 10.1215/S1152851703000620 15279715 PMC1871999

[2] Ostrom QT Bauchet L Davis FG Deltour I Fisher JL Langer CE. The epidemiology of glioma in adults: a state of the science review. Neuro Oncol. (2014) 16:896–913. 10.1093/neuonc/nou087 24842956 PMC4057143

[3] Upadhyay N Waldman AD. Conventional MRI evaluation of gliomas. Br J Radiol. (2011) 84:S107–11. 10.1259/bjr/65711810 22433821 PMC3473894

[4] Kao HW Chiang SW Chung HW Tsai FY Chen CY. Advanced MR imaging of gliomas: an update. Biomed Res Int. (2013) 2013:970586. 10.1155/2013/970586 23862163 PMC3686060

[5] Shergalis A Bankhead A Luesakul U Muangsin N Neamati N. Current challenges and opportunities in treating glioblastoma. Pharmacol Rev. (2018) 70:412–45. 10.1124/pr.117.014944 29669750 PMC5907910

[6] O'Duibhir E Carragher NO Pollard SM. Accelerating glioblastoma drug discovery: convergence of patient-derived models, genome editing and phenotypic screening. Mol Cell Neurosci. (2017) 80:198–207. 10.1016/j.mcn.2016.11.001 27825983 PMC6128397

[7] Dhermain FG Hau P Lanfermann H Jacobs AH van den Bent MJ. Advanced MRI and PET imaging for assessment of treatment response in patients with gliomas. Lancet Neurol. (2010) 9:906–20. 10.1016/S1474-4422(10)70181-2 20705518

[8] Eikermann M Holzmann N Siering U Rüther A. Tools for assessing the content of guidelines are needed to enable their effective use - a systematic comparison. BMC Res Notes. (2014) 7:853. 10.1186/1756-0500-7-853 25427972 PMC4258382

[9] Brouwers MEA Appraisal of Guidelines for Research & Evaluation II AGREE. Agree Next Steps Consortium (2009).

[10] Brouwers MC Kho ME Browman GP Burgers JS Cluzeau F Feder G. AGREE II: advancing guideline development, reporting and evaluation in health care. J Clin Epidemiol. (2010) 63:1308–11. 10.1016/j.jclinepi.2010.07.001 20656455

[11] Siering U Eikermann M Hausner E Hoffmann-Eßer W Neugebauer EA. Appraisal tools for clinical practice guidelines: a systematic review. PLoS ONE. (2013) 8:e82915. 10.1371/journal.pone.0082915 24349397 PMC3857289

[12] Sardanelli F Bashir H Berzaczy D Cannella G Espeland A Flor N. The role of imaging specialists as authors of systematic reviews on diagnostic and interventional imaging and its impact on scientific quality: report from the EuroAIM Evidence-based Radiology Working Group. Radiology. (2014) 272:533–40. 10.1148/radiol.14131730 24738613

[13] Messina C Bignotti B Tagliafico A Orlandi D Corazza A Sardanelli F. A critical appraisal of the quality of adult musculoskeletal ultrasound guidelines using the AGREE II tool: an EuroAIM initiative. Insights Imaging. (2017) 8:491–7. 10.1007/s13244-017-0563-4 28755330 PMC5621989

[14] Messina C Bignotti B Bazzocchi A Phan CM Tagliafico A Guglielmi G. A critical appraisal of the quality of adult dual-energy X-ray absorptiometry guidelines in osteoporosis using the AGREE II tool: an EuroAIM initiative. Insights Imaging. (2017) 8:311–17. 10.1007/s13244-017-0553-6 28432574 PMC5438319

[15] Romeo V Stanzione A Cocozza S Ugga L Cuocolo R Brunetti A. A critical appraisal of the quality of head and neck cancer imaging guidelines using the AGREE II tool: a EuroAIM initiative. Cancer Med. (2018) 8:209–15. 10.1002/cam4.1933 30575332 PMC6346224

[16] Doniselli FM Zanardo M Manfrè L Papini GDE Rovira A Sardanelli F. A critical appraisal of the quality of low back pain practice guidelines using the AGREE II tool and comparison with previous evaluations: a EuroAIM initiative. Eur Spine J. (2018) 27:2781–90. 10.1007/s00586-018-5763-1 30220040

[17] AGREE Next Steps Consortium The AGREE ll Instrument (2017).

[18] Byrne J Dwivedi R Minks D Tumors of the brain. In: Nicholson T editor. Recommendations for Cross-Sectional Imaging in Cancer Management, Second edition London: The Royal College of Radiologists (2014).

[19] Ellingson BM Bendszus M Boxerman J Barboriak D Erickson BJ Smits M. Consensus recommendations for a standardized Brain Tumor Imaging Protocol in clinical trials. Neuro Oncol. (2015) 17:1188–98. 10.1093/neuonc/nov095 26250565 PMC4588759

[20] Essig M Anzalone N Combs SE Dörfler A Lee SK Picozzi P. MR imaging of neoplastic central nervous system lesions: review and recommendations for current practice. Am J Neuroradiol. (2012) 33:803–17. 10.3174/ajnr.A2640 22016411 PMC7968800

[21] Fouke SJ Benzinger T Gibson D Ryken TC Kalkanis SN Olson JJ. The role of imaging in the management of adults with diffuse low grade glioma: a systematic review and evidence-based clinical practice guideline. J Neurooncol. (2015) 125:457–79. 10.1007/s11060-015-1908-9 26530262

[22] Mukundan S Holder C Olson JJ. Neuroradiological assessment of newly diagnosed glioblastoma. J Neurooncol. (2008) 89:259–69. 10.1007/s11060-008-9616-3 18712280

[23] Ryken TC Aygun N Morris J Schweizer M Nair R Spracklen C. The role of imaging in the management of progressive glioblastoma: a systematic review and evidence-based clinical practice guideline. J Neurooncol. (2014) 118:435–60. 10.1007/s11060-013-1330-0 24715656

[24] Thust SC Heiland S Falini A Jäger HR Waldman AD Sundgren PC. Glioma imaging in Europe: a survey of 220 centres and recommendations for best clinical practice. Eur Radiol. (2018) 28:3306–17. 10.1007/s00330-018-5314-5 29536240 PMC6028837

[25] Tian H Gou Y Pan Y Li Q Wei D Wang Z Quality appraisal of clinical practice guidelines on glioma. Neurosurg Rev. (2015) 38:39–47. 10.1007/s10143-014-0569-z 25199810

[26] Kim W Novotna K Amatya B Khan F. Clinical practice guidelines for the management of brain tumours: a rehabilitation perspective. J Rehabil Med. (2018) 51:89–96. 10.2340/16501977-2509 30483721

[27] Hoffmann-Eßer W Siering U Neugebauer EAM Brockhaus AC McGauran N Eikermann M. Guideline appraisal with AGREE II: online survey of the potential influence of AGREE II items on overall assessment of guideline quality and recommendation for use. BMC Health Serv Res. (2018) 18:143. 10.1186/s12913-018-2954-8 29482555 PMC5828401

